# Effects of Limbs’ Spasticity on Spinopelvic Alignment in Post-Stroke Patients: A Cross-Sectional Study

**DOI:** 10.3390/jcm13133840

**Published:** 2024-06-29

**Authors:** Luciano Bissolotti, Alice Brojka, Marika Vezzoli, Stefano Calza, Federico Nicoli, Carlos Romero-Morales, Jorge Hugo Villafañe

**Affiliations:** 1 Fondazione Teresa Camplani Casa di Cura Domus Salutis, 25123 Brescia, Italy; luciano.bissolotti@fondazionecamplani.it (L.B.); federico.nicoli@fondazionecamplani.it (F.N.); 2 Physical Medicine & Rehabiltation School of Specialty, University of Pavia, 27100 Pavia, Italy; alice.brojka@fondazionecamplani.it; 3 Department of Molecular and Translational Medicine, University of Brescia, 25123 Brescia, Italy; marika.vezzoli@unibs.it (M.V.); stefano.calza@unibs.it (S.C.); 4 Department of Physiotherapy, Faculty of Sport Sciences, Universidad Europea de Madrid, 28670 Villaviciosa de Odón, Spain; carlos.romero@universidadeuropea.es; 5 Musculoskeletal Pain and Motor Control Research Group, Faculty of Sport Sciences, Universidad Europea de Madrid, 28670 Villaviciosa de Odón, Spain

**Keywords:** stroke, spasticity, spine

## Abstract

**Objectives**: This study aimed to determine the impacts of upper and lower limb (UL and LL) spasticity and impairment on spinal alignment in chronic post-stroke patients. **Methods**: A total of 45 consecutive chronic post-stroke patients, 18 women and 27 men, from 18 to 70 years old who presented post-stroke hemiparesis were recruited in this cross-sectional study. The clinical assessment included the Modified Ashworth Scale (UL-MAS and LL-MAS spasticity), Upper Limb Motricity Index (UL-MI), FAST-UL, and Five Times Sit-to-Stand Test (5T-STS); the Associated Reaction Rating Scale was used to measure associated reactions in the hemiparetic UL, the plumb line distance from the spinous process of C7 on the sagittal (PL-C7s) and frontal plane (Pl-C7f), the kyphosis apex (PL-AK), and the spinous process of L3 (PL-L3). Angular measures of spinal alignment were measured by a Bunnell scoliometer™ (angle of trunk rotation—ATR) and a gravity-dependent inclinometer (inclination at C7-T1 and T12-L1). **Results**: In chronic post-stroke patients, there was found to be an association between the 5T-STS and PL-C7f (β = 0.41, *p* = 0.05) and the angle of inclination at T12-L1 (β = 0.44, *p* = 0.01). The FAST-UL correlated with PL-C7f (β = −0.41, *p* = 0.05), while the UL-MI correlated with this last parameter (β = −0.36, *p* = 0.04) and the ATR (β = −0.31, *p* = 0.05). The UL-MAS showed correlation with the ATR (β = 0.38, *p* = 0.01). **Conclusions**: The results lead to the possibility that, in chronic post-stroke patients, spinal misalignment on the frontal and sagittal plane is associated both with strength impairment and UL spasticity. The improvement or restoration of spinopelvic parameters can take advantage of therapeutic interventions targeted at motor improvement and spasticity reduction of the hemiparetic side.

## 1. Introduction

Stroke stands as the second leading cause of death and the third leading cause of death and disability combined worldwide, as measured by disability-adjusted life years (DALYs). The global cost of stroke exceeds USD 721 billion, constituting 0.66% of the global GDP. Between 1990 and 2019, there was a significant increase in stroke burden, with a 70.0% rise in incident strokes, 43.0% rise in stroke-related deaths, 102.0% rise in prevalent strokes, and 143.0% rise in DALYs. The majority of this burden, comprising 86.0% of deaths and 89.0% of DALYs, is observed in lower-income and lower-middle-income countries. In Italy in 2019, the National Register documented 86,360 hospital admissions following a stroke [1]. The mortality rate at 30 days from the onset ranges from 20 to 30%, increasing to 40–50% after one year. Of the survivors, 75% experience some form of disability, half of which lead to a loss of self-independence [2]. Stroke poses a significant challenge to national health systems due to its prevalence and its disabling impact on individuals and their families.

In stroke survivors, upper limb impairment disrupts intricate hand and arm grasping functionalities [3]. Stroke-related consequences include muscle weakness, defined by the inability to activate specific upper limb muscles or segments, and altered inter-joint coordination, involving the spatial and temporal control of all upper limb joints or segments [4]. Phenotypes of stroke-related inter-joint coordination deficits manifest as pathological flexor synergy during reaching, increased trunk movements compensating for upper limb limitations, and decreased finger dexterity for prehensile grasp application. 

The pathological flexor synergy, characterized by stereotypical co-activation of elbow flexion and shoulder abduction, is evident in reaching, arm load-related reductions in the upper limb workspace and diminished finger extension [5,6,7]. Trunk movements during reaching are considered compensatory strategies for upper limb motor impairments, with associations to the level of impairment [8]. These movement abnormalities may appear in isolation or combination, persisting chronically based on deficit severity and the affected cerebral region, which poses an ongoing challenge for treatment approaches [9].

As per the International Classification of Functioning, Disability, and Health model [10], impairments may be categorized as deviations or losses in neuromusculoskeletal and movement-related functions (body function) or significant deviations in structures related to movement (body structures) [10]. Stroke can result in both types of impairments [10]. Understanding upper limb impairments is complex due to their dynamic nature and the potential coexistence of multiple impairments over time. 

Weakness or paralysis is the primary impairment contributing to dysfunction after stroke, while spasticity, characterized by a velocity-dependent increase in muscle tone with exaggerated tendon jerks, is a component of upper motor neuron syndrome (UMNS) [11].

The symptoms of spasticity can vary widely depending on the underlying cause, the specific muscles affected, and the severity of the condition. Common symptoms include increased muscle stiffness and involuntary muscle contractions, while clonus, pain, and discomfort are other signs and symptoms of UMNS [12]. Consequently, post-stroke patients present difficulty with movement and have a high risk of developing joint deformities in the upper and lower limb affected by spasticity. Postural abnormalities and fatigue impair the ability of patients to independently and safely perform daily life activities [13]. As is frequently observed in clinical settings, the different localizations of spasticity can determine various forms of motor impairment in post-stroke patients. In the lower limb, the presence of a spastic equinovarus foot can result in an asymmetric step cycle and a balance impairment with an increased risk of falling, while in the upper limb, the spasticity of the flexor muscles may result in failure to perform reaching tasks or grasp objects with the hand [13,14]. 

The prevalence of spasticity increases with time since stroke and is linked to the secondary effects of weakness and immobility on skeletal muscles [15,16].

In stroke-affected patients, the control of trunk, pelvis, and leg muscles is crucial for maintaining the center of mass within a stable base of support [17]. Examining trunk position sense can provide insights into trunk alignment with gravity, and sitting lateral balance control, critical for post-stroke patients, is often affected [18]. Despite the interest in correlations between upper limb and lower limb spasticity and trunk posture, there is limited research on these relationships [19]. Because spinopelvic alignment is a key parameter in this study, it is important to elaborate on its definition and typical treatments. Spinopelvic alignment refers to the relationship between the spine and the pelvis in the sagittal plane. It is essential for maintaining an upright posture and balance. Key parameters include pelvic tilt (PT), pelvic incidence (PI), and sacral slope (SS). PT is the angle between the vertical and the line connecting the center of the femoral heads with the midpoint of the sacral plate. PI is an anatomical parameter that describes the shape of the pelvis and directly affects PT and lumbar lordosis. SS is the angle between the sacral plate and the horizontal, influencing lumbar lordosis directly [20]. Treatment for spinopelvic alignment can be conservative or surgical. Conservative management includes physical therapy and specific exercises to strengthen back muscles and improve flexibility. Orthoses can also help maintain proper posture and reduce pain. In severe cases where conservative treatment fails, surgical interventions such as corrective osteotomy and spinal fusion are necessary to restore sagittal alignment and improve the patient’s quality of life [21]. Our study investigates the influence of upper and lower limb performance and spasticity on frontal, sagittal, and horizontal displacement of the trunk in chronic post-stroke patients.

## 2. Materials and Methods

This cross-sectional study was conducted at the Clinic Fondazione Teresa Camplani Casa di Cura Domus Salutis (Brescia, Northern Italy) between November 2022 and June 2023. A total of 49 consecutive chronic post-stroke patients, 18 women and 31 men, from 20 to 70 years old who presented post-stroke hemiparesis were recruited during medical examination in the physical therapy department of the clinic. The causes of stroke were ischemia in 75% of patients and hemorrhage in 25% of patients; 62% of patients had a right-sided lesion. At the time of onset of acute stroke, the diagnosis was performed by computed tomography or a magnetic resonance imaging scan. The study was limited to patients in the chronic phase of stroke (more than 6 months after the onset of acute stroke). The inclusion criteria included preserved cognitive capacities according to age (Mini-Mental State Examination score > 24), primary ischemic or hemorrhagic stroke, mild to moderate paresis of the upper limb, and absence of pain on movement [22]. The Modified Ashworth Scale (MAS) is a widely used clinical tool for assessing the degree of spasticity and provides a standardized way to measure muscle tone, helping clinicians to evaluate the severity of spasticity and monitor changes over time or in response to treatment. The Modified Ashworth Scale grades spasticity on a scale from 0 to 4. The exclusion criteria included subjects that had a spasticity score of greater than three on the Modified Ashworth Scale (MAS = 4: affected part(s) rigid in flexion or extension) [23], a pain score greater than six (severe pain) on the Visual Analogue Scale (VAS) at the upper or lower limb affected by spasticity [24], presence of finger flexion contracture, earlier or known diagnosis of scoliosis (adolescent idiopathic or degenerative scoliosis of adult age), leg length discrepancy higher than 10 mm, degenerative or non-degenerative neurological conditions in which pain perception could be altered, presence of persistent orthostatic hypotension (defined as a drop in blood pressure greater than 30 mmHg in sit-to-stand passage), and a Body Mass Index greater than 34. Participants with apraxia, anxiety, or major depressive disorders were also excluded. Informed consent was obtained from all participants, and all procedures were conducted according to the Declaration of Helsinki. The study was approved by the Ethical Committee AST Brescia (No. NP 5754).

### 2.1. Clinical Measurements

At the beginning of the clinical examination, we recorded the age, sex, date of onset, type, and side of acute stroke. Afterwards, subjects underwent a clinical examination including body weight and standing height assessment as well as Body Mass Index calculation. In the affected side, upper limb and lower limb spasticity were assessed according to the MAS criteria [25]. In the UL, according to the MAS criteria, we evaluated shoulder abduction, elbow extension, forearm supination, wrist extension, and finger extension, while in the LL we measured the spasticity of hip flexors, hip abductors, knee extensors, and ankle plantar flexors. The Motricity Index upper limb scoring subset was used to measure the strength of shoulder abductors, elbow flexors, and pinching [26], while the FAST-UL was used to provide a Functional Assessment Test for Upper Limb performance based on the observational analysis performed by the examiner [27]. The Associated Reaction Rating Scale was used to measure associated reactions in the hemiplegic upper limb as described by Macfarlane et al. [28]. The Five Times Sit-to-Stand Test measured one of the aspects of transfer skills to provide an objective assessment of patients’ mobility [29].

This first phase of the assessment procedure was followed by a spinal alignment evaluation by sagittal arrow determination at the levels of C7 (sagittal and frontal plane), the kyphosis apex, L3, and S1. The apex of the kyphosis was used as the reference, and the distance to that level was consequently subtracted from all other measurements as previously described by Zaina et al. [30,31]. Patients were categorized as being affected by hypolordosis if the distance of L3 measured from the plumb line was below 25 mm and as affected by hyperkyphosis if the sagittal index obtained from the sum of the plumb line distance from C7 and L3 exceeded 95 mm. This value being exceeded was previously shown to be correlated with a higher probability of identifying hyperkyphosis during X-ray examination [32]. The angle of trunk rotation (ATR, expressed in degrees) was also measured with a Bunnell scoliometer™ (Orthopedic Systems, Inc., Hayward, CA, USA) in a standing, bent-over position (arms dangling) [20,21]. The sagittal profile of the spine was measured by inclination assessment with a gravity-dependent inclinometer (Isomed Inc., 975 SE Sandy Blvd, Portland, OR, USA) [33]. All measurements were performed by the same operator participating in the general practitioner consultations and took approximately 30 s. The measurement errors were previously reported in other studies [32].

### 2.2. Statistics

**Figure 1:**
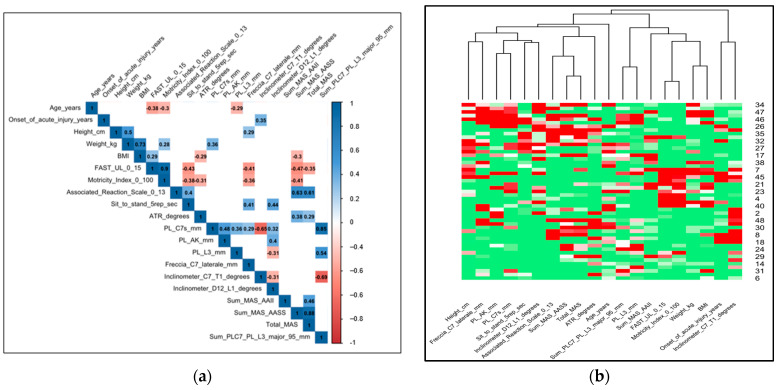
(**a**,**b**): Correlation plot with all the quantitative variables. This upper triangular matrix reports in each cell the spearman correlation ($\rho_{s}$) coefficient computed between couples of items. The background of the cells is coloured of blue if the relationship is positive (${0\leq \rho }_{s}\leq 1$); otherwise, cell is coloured of red (${-1\leq \rho }_{s}<0$). The colors intensity are proportional to the magnitude of ($\rho_{s}$), white cells corresponds to correlations which are not significant different from 0 (*p*-value > 0.05).

The following descriptive statistics were computed for quantitative variables in the dataset: mean, standard deviation (SD), median, first (Q1) and third (Q3) quartile, and range (minimum–maximum). Spearman correlation coefficients (*ρs*) between pairs of quantitative variables were visualized by means of a correlation plot since this is an easy and intuitive way to show relationships between the data. This graph shows an upper triangular matrix where blue and red cells correspond to positive (0 ≤ *ρs* ≤ 1) and negative (−1 ≤ *ρs* < 0) correlations, respectively. Moreover, the color intensity is proportional to the magnitude of the Spearman indexes (the value of which is reported in each cell). White cells correspond to non-significant correlations (in other words, the correlation test *p*-values are greater than 0.05). Following the visual mining philosophy, a heatmap was used (Figure 1a,b). This is a graphical representation of the quantitative data collected for the analysis. Starting from the standardized data matrix (each variable has a mean = 0 and variance = 1), this graph inserts statistical units (patients) in rows and variables in columns. Each cell is colored based on the magnitude of the corresponding value (green, white, and red shades correspond to values under, around, and above the mean, respectively). In the graph, similar patients and variables are placed near each other according to the complete agglomerative hierarchical cluster analysis where the distance matrix is obtained by applying the Euclidean measure (Figure 1a,b). To easily identify variables with a similar trend, the graph shows the dendrogram in columns. All the analyses were performed using R version 4.2.1 (packages: arsenal, corrplot, ppcor, gplot, vegan).

## 3. Results

### 3.1. Demographic Characteristics

A total of forty-nine post-stroke patients were enrolled in the study, with 40% being women and a median age of 58.1 years. Left hemiparesis was prevalent in 62% of the participants, and 75% attributed their hemiparesis to ischemic stroke (Table 1). Further demographic details and functional scores for upper and lower limb performances are outlined in Table 2, while Table 3a,b provide insights into the severity of spasticity in different joints using the Modified Ashworth Scale.

### 3.2. Functional Task Associations

The Five Times Sit-to-Stand (5T-STS) performance exhibited a significant association with the frontal plane parameter plumb line values at C7 in the frontal plane (PL-C7f) (β = 0.41, *p* = 0.05) and the angle of inclination at T12-L1 (β = 0.44, *p* = 0.01). Moreover, the FAST-UL demonstrated a correlation with PL-C7f (β = −0.41, *p* = 0.05). Additionally, the UL-MI was correlated with both PL-C7f (β = −0.36, *p* = 0.04) and the ATR (β = −0.31, *p* = 0.05), underlining the multifaceted nature of these associations. The UL-MAS also showed a correlation with the ATR (β = 0.38, *p* = 0.01), emphasizing the relevance of trunk alignment in upper limb motor assessment.

## 4. Discussion

Given the elevated risk of both short-term and long-term complications following a stroke, this study explored spinopelvic alignment and analyzed correlations between motor performance, spasticity on the hemiparetic side, and specific parameters in the sagittal, frontal, and horizontal planes. The primary objective was to enhance rehabilitation strategies and conservative management of trunk neurogenic deformities identified in post-stroke patients with spasticity [34]. To the best of our knowledge, this study represents the first investigation into the strength of the association between post-stroke consequences and spinal alignment as measurable during a clinical examination. Previous research has examined spinal maladaptive changes in various neurological and musculoskeletal pathologies, establishing associations with disease severity and disability, though not specifically in chronic post-stroke patients [21,30,35]. 

In this cohort of post-stroke spasticity patients, significant correlations were observed among several parameters. The ATR, PL-C7f, and thoracolumbar inclination (inc. D12-L1) were found to be influenced by the severity of upper limb spasticity and performance (Table 3). Notably, this study revealed that upper limb spasticity primarily affects trunk posture in the horizontal plane, with a higher severity of spasticity correlating with increased ipsilateral rotation of the trunk, as indicated by the angle of trunk rotation (ATR Bunnell).

This study’s results demonstrated that the Bunnell scoliometer-measured vertebral rotation (ATR) in the horizontal plane may serve as a morphological indicator of scoliosis in post-stroke patients during routine medical examinations. Additionally, correlations were found between PL-C7f and the Five Times Sit-to-Stand Test, with negative correlations observed with upper limb global motor performance assessed through the Motricity Index and FAST-UL.

The observed positive correlation between PL-C7f and the Five Times Sit-to-Stand Test, along with negative correlations with upper limb motor performance, suggests that PL-C7f can serve as a marker of trunk imbalance in the presence of scoliotic deformity. These findings align with biomechanical considerations, as there is a logical correlation between the Five Times Sit-to-Stand Test and lateral displacement of the spine. In post-stroke patients, impaired strength and standing balance are common, contributing to postural decompensation on the frontal plane during activities such as sitting to standing.

Neurophysiological mechanisms, including asymmetric lower limb performance during sit-to-stand, were also explored. This study posits that interventions aimed at restoring trunk alignment in patients with upper motoneuron syndrome should encompass strength and balance exercises complemented by pharmacological treatments to enhance muscle recruitment on the paretic side during various static or dynamic exercises [36,37]. 

In our study, we observed a negative correlation between two fundamental ICF domains of upper limb performance: strength, measured by Motricity Index sub-scores (body function domain), and the ability to move the arm in space, measured by the FAST-UL. A diminished ability to recruit muscles and move the arm increases the likelihood of presenting a lateral shift of the trunk in the frontal plane. Once again, we contend that any intervention aimed at improving upper limb motor function has the potential to mitigate trunk imbalance in the frontal plane.

### Limitations

Several limitations should be acknowledged in this study. The heterogeneity in patient age (range: 20 to 77 years) and disease duration (range: 6 months to 30 years) prevented a reliable analysis of spinal sagittal balance. However, the exclusion criteria ensured the absence of degenerative scoliosis in the patient cohort, mitigating the impact of age on the results. Additionally, a mild prevalence of left hemiparesis (62%) may have influenced the results due to potential impairments in vision and spatial exploration on the ipsilateral side.

## 5. Conclusions

While X-ray examination remains the gold standard for spine evaluation, this study proposes non-radiographic methods, such as plumb line distances and the Bunnell scoliometer, as valuable tools for assessing spinal imbalance in chronic post-stroke patients without specific symptoms or clinical signs justifying exposure to ionizing radiation. The Five Times Sit-to-Stand Test has emerged as a marker of global performance and better trunk control in the frontal plane. This study provides a cost-effective protocol for trunk balance assessment in post-stroke patients, offering a practical approach for clinicians that can be easily implemented in a clinical setting. This protocol holds potential for application in larger studies exploring trunk posture changes in post-stroke patients or investigations focusing on improving sagittal balance and trunk posture as primary clinical rehabilitation targets.

## Figures and Tables

**Table 1:** Descriptive statistics of hemiparetic patients.

	Hemiparetic Patients (*n* = 49)		
	Median	Q1	Q3
Age (years)	58.1	48.4	63.6
Height (cm)	170.0	166.0	175.0
Weight (kg)	73.0	67.5	78.0
BMI	24.6	22.9	27.2
Onset of acute injury (years)	4.5	2.1	11.1
	N	%	
Left hemiparesis (N/%)	28	62%	-
Sex (F)	18	40%	
Ischemic	34	75%	-

BMI: Body Mass Index.

**Table 2:** Functional scores of hemiparetic patients.

	Hemiparetic Patients (*n* = 49)		
	Median	Q1	Q3
FAST-UL:			
Reaching (0–3)	1	1	2
Hand to mouth (0–3)	2	1	2
Supination (0–3)	0	0	1
Pinch (0–3)	0	0	1
Grasp (0–3)	0	0	1
Total score (0–15)	3	1	8
UL-Motricity Index:			
1-Shoulder abduction (0–33)	14.0	9.0	19.0
2-Elbow flexion (0–33)	14.0	9.0	22.0
3-Pinching (0–33)	0.0	0.0	12.5
Total score (0–100)	29.0	19.0	56.0
Associated Reaction Rating Scale (0–13)	6.0	4.0	7.5
Five Times Sit-to-Stand (sec)	17.5	14.0	21.7

FAST-UL: Functional Assessment Test for Upper Limb; UL-Motricity Index: Upper Limb Motricity Index scores.

**Table 3:** (**a,b**). Distribution of Modified Ashworth Scale upper (a) and lower (b) limb scores in hemiparetic patients.

a.		Modified Ashworth Scale (0–4):								
	Shoulder Abduction		Elbow Extension		Supination		Wrist Extension		Finger Extension	
MAS Category	N	%	N	%	N	%	N	%	N	%
0–1	36	80%	25	56%	26	58%	29	64%	28	62%
2	7	16%	16	36%	7	16%	7	16%	7	16%
3	2	4%	4	9%	12	27%	8	18%	10	22%
4	0	0%	0	0%	0	0%	1	2%	0	0%
**b.**		**Modified Ashworth Scale (0–4):**								
	**Hip Flexion**		**Hip Abduction**		**Knee Flexion**		**Ankle Extension**			
MAS Category	N	%	N	%	N	%	N	%		
0–1	42	93%	43	96%	44	98%	29	64%		
2	3	7%	2	4%	0	0%	12	27%		
3	0	0%	0	0%	0	0%	2	4%		
4	0	0%	0	0%	0	0%	2	4%		

MAS: Modified Ashworth Scale.

## Data Availability

Data are contained within the article.
